# An Improved Chemical Extraction Procedure for the Sr Isotope Analysis of Liquid Agrifood Samples Applied to Authenticating the Origin of Maple Syrups in Quebec (Canada)

**DOI:** 10.1002/rcm.10066

**Published:** 2025-05-13

**Authors:** B. Saar de Almeida, R. Stevenson, M. Sadiki, L. Lagacé, D. Widory

**Affiliations:** ^1^ Université du Québec à Montréal (UQAM)/GEOTOP Montréal Quebec Canada; ^2^ Centre de recherche, de développement et de transfert technologique acéricole Inc. (Centre ACER) St‐Norbert d'Arthabaska Quebec Canada

**Keywords:** food traceability, maple syrup, Quebec, soils, strontium isotopes

## Abstract

**Rationale:**

We describe a new simplified sample preparation technique, based on dual chromatography for the analysis of ^87^Sr/^86^Sr in liquid agri‐food samples. We applied this approach for authenticating the origin of maple syrup products in Quebec, characterizing the ^87^Sr/^86^Sr of soil profiles from geologically distinct maple groves, including maple tree components and maple syrup products.

**Methods:**

In our simplified technique, 3 mL of the organic liquids was poured into a 15‐mL centrifuge tube, diluted with 10 mL of Mill‐Q H_2_O, and manually shaken to produce a thin juice. The thin juice was subsequently loaded in two 7.5 mL aliquots onto 10‐mL Bio‐RadTM chromatography columns containing 4 mL of cleaned AG50‐X8 resin (100–200 mesh in a 1‐N HCl solution). Once the sample was absorbed by the resin, the organic fraction of the thin juice was eluted by adding 3 × 5 mL of 1‐N HCl. The Sr and other cations were subsequently recovered by adding 2 × 5 mL of 6‐N HCl.

**Results:**

The maple groves and the ^87^Sr/^86^Sr ratios for maple syrup from 39 different producing areas in Quebec indicated that no isotope fractionation occurs between the syrup, the maple trees, and the corresponding labile fraction of the soil they grew upon. This suggests that ^87^Sr/^86^Sr provides a reliable isotope fingerprint for the provenance of maple syrups. Gathering available agri‐food ^87^Sr/^86^Sr data across the Quebec province, we constructed the first bioavailable ^87^Sr/^86^Sr map based on actual agri‐food data.

**Conclusions:**

This study reported an improved method for Sr separation for ^87^Sr/^86^Sr studies in liquids by combining two different cation‐exchange chromatography steps. Data were used to develop a bioavailable ^87^Sr/^86^Sr map that can be used to predict the geographical origin of agrifood products from southern Quebec.

## 1 Introduction

Maple syrup is one of Canada's most important non‐timber forest products (NTFPs). The province of Quebec accounts for 91% of the Canadian production (more than 11 million gallons by year) representing the world's largest and most prestigious producer of maple syrup (75% of the world's production). Maple syrup is produced from the sap collected from maple trees ( *Acer saccharum* ) in the early spring. During the spring thaw, the difference in night‐time (−5°C to −10°C) and daytime (5°C to 10°C) temperatures creates pressure inside the tree that enables the sap to flow through a collection system. The sap is collected and concentrated by boiling (water evaporation) or by reverse osmosis followed by evaporation. The concentrate mostly consists of sucrose, glucose, fructose, and trace levels of oligosaccharides, organic acids, minerals, and amino acids (e.g., [1, 2]). Calcium (Ca), magnesium (Mg), and potassium (K) represent the major metals present in maple sap and syrup [3].

The certification of food products, their authenticity, and origins is a growing priority for consumers and producers. Fingerprinting Canadian maple syrups requires the development and implementation of forensic tools to link them to their corresponding production areas or terroir. Radiogenic isotope systematics including strontium (Sr) isotopes have proven value for authenticating food provenance (e.g., [4, 5, 6]). Sr is an alkaline Earth metal, occurring in nature as Sr^2+^. It can substitute for Ca^2+^ in a variety of rock‐forming minerals such as feldspars, gypsum, plagioclase, apatite, and carbonates [3, 7]. Sr has four naturally occurring stable isotopes whose abundances range from 0.55% to 0.58% for ^84^Sr, from 9.75 to 9.99% for ^86^Sr, from 6.94 to 7.14% for ^87^Sr, and from 82.3 to 82.8% for ^88^Sr [8]. ^84^Sr, ^86^Sr, and ^88^Sr isotopes occur in relatively stable percentages, whereas ^87^Sr gradually increases through ^87^Rb radioactive decay. Thus, ^87^Sr/^86^Sr mainly depends on the nature and age of the host rock: Older rocks and higher Rb contents yield more radiogenic ^87^Sr/^86^Sr ratios due to the prolonged production of ^87^Sr due to ^87^Rb decay.

Radiogenic isotope systematics such as Sr isotopes are increasingly used to authenticate the geographical origin of numerous agri‐food products (wine, milk, ciders, e.g., [9, 10, 11]). The underlying theory is that Sr concentrations and ^87^Sr/^86^Sr isotope ratios in agri‐food products are derived from the soil on which they grew. More precisely, the Sr absorbed by the plants is derived from the labile fraction (water/organic acid soluble portion) of the soil, and the chemical extraction of the labile fraction has been described elsewhere (e.g., [5, 12, 13]). The application of Sr isotopes to provenance studies requires the analysis of a high number of samples, and thus, rapid sample digestion, chemical separation, and analytical procedures are desired. Agri‐food products pose a problem for the rapid digestion of the samples due to the high content of organic material that leaves a black, relatively insoluble residue when attacked by mineral acids such as hydrochloric, nitric, and/or hydrofluoric acids. Removal/digestion of the organic material requires time‐consuming digestion of the sample via heat and/or repeated application of oxidizing reagents (nitric acid and/or peroxide; e.g., [5]).

We propose a simplified sample preparation technique that removes the need for sample heating or digestion/oxidation and that can be applied to most organic‐based liquids prior to Sr purification.

This purification technique was tested using maple syrup from different maple grooves from Quebec, Canada. These analyses are supported by soil profiles from two different maple syrup production areas. The selected samples span 3 different geological domains with well‐defined ages: the Grenville province (from Archean to Mesoproterozoic), the St. Lawrence Platform (upper and middle Ordovician), and the Appalachian Orogen (Cambrian and lower Ordovician).

This paper has three objectives: (i) test a simplified purification technique for high‐sugar agrifood beverages, by analyzing samples of maple syrup using MC‐ICP‐MS and TIMS; (ii) constraining the Sr isotope relationship between the soil, plant, sap, sap concentrate, and ultimately the maple syrup, focusing specifically on determining if the Sr isotope ratios are preserved during absorption from the soil and across the evaporation cycles; (iii) authenticating the origin of maple syrups using Sr isotope systematics coupled with a geological map of Quebec (1:2000.000, [14]).

Houle et al. [15] recently investigated the link between the ^87^Sr/^86^Sr ratios of sugar maple's tree rings and soil samples in 3 different places in Quebec, but, to our knowledge, strontium isotope ratios in maple syrup have never been tested. The maple syrup analyses are compared with previous Sr isotope studies of grapes, wine, milk, and cheese from the same geological provinces within Quebec [5, 11, 12].

## 2 Methods

### 2.1 State of Art

The Sr isotope tracing technique was developed by geoscientists to date the age of igneous rocks (geochronology) and trace the origin of geological reservoirs (crust vs. mantle) that gave rise to the rock in question. Sample preparation of rock samples typically involves crushing the rock to obtain a representative powder, followed by acid digestion of the rock to obtain a solution containing the Sr. The Sr is subsequently purified by ion exchange chromatography and then the isotope composition is analyzed via mass spectrometry. Rock dissolution by acid digestion is relatively straightforward involving either HCl or HNO_3_ (carbonates) or a combination of HF and HNO_3_ (silicates).

The digestion of wine, maple syrup, or honey typically involves the addition of a combination of nitric acid (HNO_3_) and peroxide (H_2_O_2_) to digest the organic material. For example, [16] studied the trace element geochemistry of maple syrup from Nova Scotia. Their sample preparation began with oxidizing the maple syrup with 10% HNO_3_ for 24 h, followed by additional attacks of concentrated HNO_3_ and H_2_O_2_ to eventually obtain a clear liquid for ICP‐MS concentration analysis. Similar digestion recipes have been used to obtain organic‐free liquids for Sr isotopes in wine and cider [5, 9, 12, 17, 18, 19]. The method often requires repeated applications of either HNO_3_ and/or peroxide.

Microwave digestion of organic samples such as wine and honey using nitric acid and H_2_O_2_ has been shown to speed up the digestion and oxidation but requires specialized microwave ovens and containers (e.g., [9, 20]). Alternatively, [21] used ultraviolet radiation and peroxide to decompose organic material in diluted wine samples prior to cation exchange chromatography. Durante et al. [22] proposed a simplified chemical protocol for extracting Sr from wine that involved equilibrating wine samples with concentrated nitric acid over a 12‐h period that resulted in a mineralized solution from which the Sr was extracted via cation exchange. The ashing technique is frequently used to reduce solid organic samples to an acid‐soluble ash and has been used to prepare liquid organic substances such as honey for Sr isotopes analyses [23]. The above sample preparation protocols involve combinations of concentrated acids that can lead to larger reagent blanks, with possible vigorous reactions between organic‐rich liquids and acids/peroxide, and often require multiple treatments to oxidize the organic material.

We describe below a simplified sample preparation protocol for organic liquids such as maple syrup, wine, or honey that removes the need for digestion reactions requiring combinations of nitric acid and peroxide. The method is based on the softening/decalcification of thin juice by cation chromatography, a technique that has been employed by sugar and fruit juice manufacturers for over 70 years. Color in sugars (cane or beet sugar) is caused by the presence of natural organic molecules as well as molecules produced during the refining process [24, 25]. The removal of unwanted color in sugar and softening of sugar (removal of K, Ca, and Fe) has been an important process in sugar refining since the early 20th century [26]. Naturally occurring Ca in the sugars also poses a problem in the refining process because evaporation of the sugar slurry during refining leads to large deposits of Ca (scaling) on the equipment [27]. The sugar industry found solutions to this problem by passing water‐diluted sugar juice (thin juice) through a cation exchange resin ([27]) that yielded Ca‐free, softened (hydrogenated) thin juice. This simple process is essentially exactly what is desired in order to isolate Sr for Sr isotope studies of agri‐food beverages.

We outline below a two‐column chromatography procedure that eliminates the need for acid digestion of liquid agrifood products. In this procedure, the sugar or beverage is diluted to form a thin juice, acidified, and loaded into a cation exchange column that traps the cations (Ca, Sr, K) in the juice. The juice and organic material are eluted, leaving behind the desired cations. The cations are subsequently eluted, and the Sr is purified via a second chromatography step.

### 2.2 Sampling Strategy

Samples from distinct producers were provided by the Centre ACER (Centre for Maple Research, Development and Technology Transfer in Quebec, Canada). The sampling sites were entered into a georeferenced database, using ArcGIS ENRI facilities, including background geology from the geological service of the government of Quebec, Canada [14].

**FIGURE 1:**
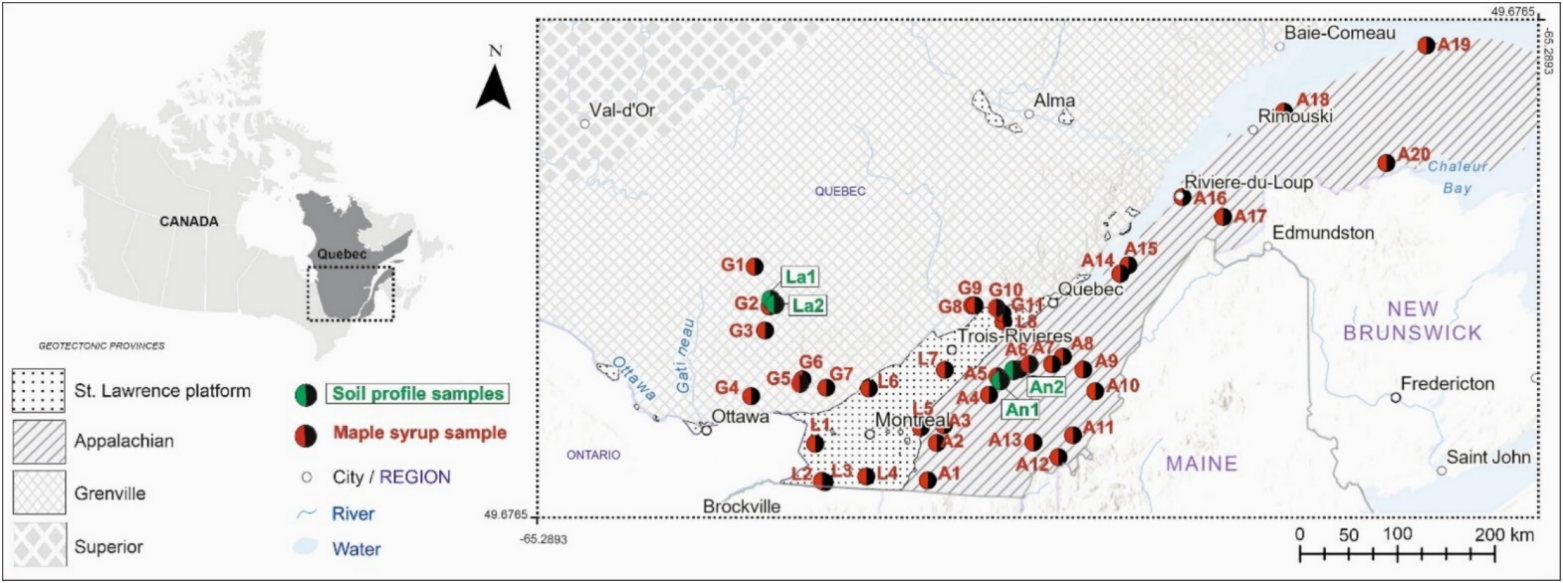
Map showing maple syrup sampling and soil profile locations and the surrounding geological provinces.

A total of 39 maple syrup samples were selected from different maple syrup production areas spanning three geological provinces of contrasting age, structure, and rock types. These are the Grenville, Appalachian, and St. Lawrence platform geological provinces (Figure 1).

The Grenville Province is largely characterized by high‐grade metamorphic orthogneiss and paragneiss complexes with protolith ages ranging from 1.0 to 2.7 Ga. The province is bounded by the Superior and Churchill provinces in the north and sedimentary rocks of the St. Lawrence Platform and the Appalachian Province to the south. Cambrian‐aged rifting associated with the opening of the Iapetus Ocean along the southeastern margin of the Canadian Shield deposited sandstones, shales, and carbonates (570–430 Ma) that characterize the St. Lawrence platform. These Cambrian to Devonian‐aged sedimentary rocks are largely flat lying and overlay the Grenville Province. The Iapetus Ocean was closed by thrusting associated with the Acadian and Taconic orogenies that formed the Appalachian Province of Southern Quebec. These Appalachian orogenies folded, faulted, and metamorphosed the volcanic and sedimentary rocks (600–300 Ma) that formed outboard of the St. Lawrence Platform [28].

**FIGURE 2:**
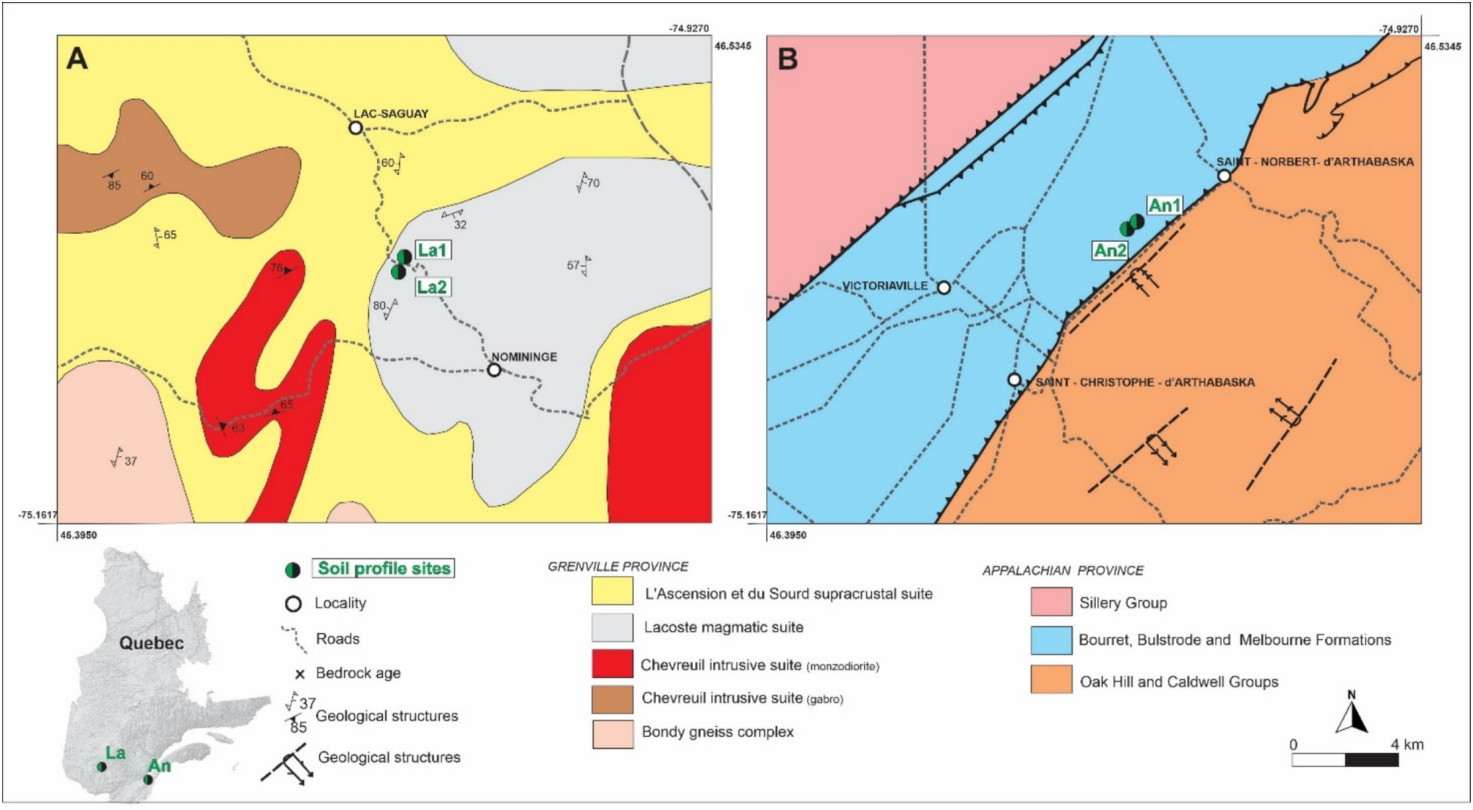
Simplified geological map of studied soil profiles in Nominingue (A) and Saint Norbert d'Arthabaska (B) areas. Based on the Quebec Geological Survey regional map [14].

A detailed study of four soil profiles was carried out in maple groves within the Grenville (Nominingue) and Appalachian (Saint Norbert d'Arthabaska) geological provinces (Figure 2). The soil profiles from the Laurentide region of the Grenville Province (La1 and La2) are underlain by rocks from the Lacoste Magmatic suite, consisting of metamorphosed plutons that are interleaved with rocks of the Ascension and du Sourd supracrustal suites [29]. The Lacoste Magmatic Suite consists of tonalite, amphibolite, gabbro, enderbite, and monzogranite units [30, 31, 32, 33, 34, 35]. The Lacoste Magmatic Suite was emplaced at 1347 Ma and subsequently metamorphosed at 1059 Ma and ~1000 Ma during the Grenville Orogeny [29, 36].

Soil profiles sampled close to the city of Saint Norbert d'Arthabaska (An1 and An2) are underlain by Ordovician to Lower Silurian bedrock of the Appalachian province, consisting of clayey limestone, slate, mudrock, sandstone, and conglomerate from the Bourret, Bulstrode, and Melbourne formations.

For each of the soil profile study areas, five different depth horizons were collected (0–10, 10–20, 20–30, 30–40, and 40–50 cm), following the course of the maple tree roots. We included sampling of coarse roots, stem, leaf, and litter leaf from tree superficies. To evaluate any potential variations related to the progression of the rising sap during the Spring season, samples of sap, sap concentrate (8°Brix for SN samples and 18°Brix for LA samples), and syrup were sampled during the following periods: early season, ¼‐season, mid‐season, ¾ season, and late season.

### 2.3 Sample Preparation

Sample preparation procedures and ^87^Sr/^86^Sr analyses (by TIMS and MC‐ICP‐MS) were carried out at the GEOTOP laboratories (Université du Québec à Montréal, Canada). Samples were prepared in a clean room (ISO 5 standard, 100 particles/ft2), and all digestions used acids prepared with subboiling distilled acids and Mill‐Q H_2_O, 18.2 MΩ cm for dilution. All plastic wares (pipette tips, centrifuge tubes, filters, and resin columns) were pre‐cleaned by soaking the plastics in 1‐N HCl for 24 h followed by soaking in milli Q water for 24 h.

The Sr was extracted from both the bulk soil and the labile fraction of the soil necessitating two different extraction protocols. The labile soil fraction was prepared using 2 g of soil sieved to < 2 mm. The soil was reacted twice with 10 mL of super pure ammonium acetate (NH_4_CH_3_CO_2_) for 8 h at room temperature. The supernatant portions were passed through 0.45‐μm polypropylene syringe filters using a 20‐mL syringe, and the solution was evaporated at 75°C in preparation for cation chromatography.

Bulk soil fraction samples were ashed in ceramic vessels for 8 h at 750°C (oven). One gram of the ashed soil was weighed into a Teflon beaker (15 mL) and dissolved with 5 mL of HF 29 N over 5 days at 85°C. The resulting solutions were evaporated and redissolved with 1 mL of 3‐N HNO_3_ at 85°C over 48 h.

Maple tree components (root, stem, leaf, leaf litter) were weighed (2 g) into a ceramic vessel and ashed in an oven for 8 h at 750°C. After cooling, samples were dissolved in Teflon beakers over a period of 24 h in a solution of 3 mL of nitric acid (3‐N HNO_3_) along with two drops of ultrapure hydrogen peroxide (30% H_2_O_2_).

In our simplified technique, 3 mL of the organic liquids (sap, sap concentrate or maple syrup) were poured into a 15‐mL centrifuge tube and diluted with 10 mL of Mill‐Q H_2_O and manually shaken to produce a thin juice. The thin juice was subsequently loaded in two 7.5‐mL aliquots onto 10‐mL Bio‐RadTM chromatography columns containing 4 mL of cleaned AG50‐X8 resin (100–200 mesh resin in a 1‐N HCl solution). Resin was cleaned by passing 5 mL of 6‐N HCl (three times), followed by 5 mL of 1 N HCl (three times). Once the sample was absorbed by the resin, the organic fraction of the thin juice was eluted by adding 15 mL of 1 N HCl (3 × 5 mL). The Sr and other cations were subsequently recovered by adding 10 mL of 6 N HCl (2 × 5 mL).

To provide validation, three samples were prepared using the protocol proposed above and the more traditional protocol in which 3 mL of sample is reacted with 16‐N HNO_3_ over a 24‐h period at 85°C followed by the addition of 2 drops of 30% H_2_O_2,_ until total digestion of organic matter [5, 12].

Regardless of the initial protocol, all samples were subsequently dried at 85°C for 24 h. In preparation for the purification of the Sr, the samples are dissolved in 1 mL of 8‐N HNO_3_ and placed in an ultrasonic bath for 10 min and then centrifuged for 10 min before loading onto the separation columns. Strontium was extracted from the samples by cation exchange chromatography using 0.25 mL of Eichrom Sr‐spec resin in a 1‐mL Bio‐RadTM column. The resin was washed using 2 mL of Mill‐Q H_2_O (one time) and 2 mL of 8 N HNO_3_ (three times). Samples were loaded (0.25 mL of sample in four times), rinsed (using 0.5 mL of 8‐N HNO_3_ for six times) and recovered (using 0.25 mL of 0.05‐N HNO_3_ for five times). At the end of the procedure, samples were dried at 85°C for 24 h. After evaporation, some maple syrup samples were split into two aliquots to compare analyses via TIMS and MC‐ICP‐MS. Samples analyzed by TIMS were dissolved in 1‐μL 16‐N HNO_3_ and loaded onto the Re‐filaments using 0.8‐μL tantalum oxide activator. Samples analyzed by MC‐ICP‐MS were dissolved using 5 mL of 2% HNO_3_ and then placed in an ultrasonic bath for 10 min before analysis.

### 2.4 Instrumentation and Measurements

For protocol validation, three maple syrup samples were analyzed via TIMS (Thermo Fisher) and multi‐collector inductively coupled plasma mass spectrometer (MC‐ICP‐MS, Nu Agilent). Although both instruments are capable of delivering the required precision (0.01%; [37], TIMS is favored for high precision analyses, while the MC‐ICP‐MS allows for more rapid data acquisition and higher sample throughput [13, 38]. The operating parameters for the instruments are given in Table 1. Samples and standards were introduced in solution mode using a CETAC Aridus II system in which the nebulizer gases (Ar, N_2_) were optimized for 1.5 L min^−1^ for Ar and 2–3 mL min^−1^ for N_2_. Potential interferences (Kr, residual Rb, Sr) were corrected by on‐peak‐zero analyses before each standard or sample analysis.

**TABLE 1:** Applied operating parameters for mass spectrometers instruments utilized for SRM‐987 standard and sample measurements.

MC‐ICP‐MS	
Reflected power	1300 W
Coolant gas (argon) flow rate	13.5 L min^−1^
Auxiliary gas (argon) flow rate	0.85 L min^−1^
Nebulizer gas (argon) pressure	30.7 psi for Sr
Detection system	6 Faraday collectors (low resolution)
Sample cone orifice	0.9 mm (nickel)
Skimmer cones orifice	0.6 mm (nickel)
Sample uptake rate	100 μL min^−1^
Transmission	650 V ppm^−1^ for Sr

TIMS	
^88^Sr signal	5.5 V

Repeated measurements (7) of the international standard NIST SRM 987 (Table 2) yielded a ^87^Sr/^86^Sr ratio of 0.71025 ± 0.00001 (2SD) for the TIMS and a 0.71024 ± 0.00001 (2SD) ratio for the MC‐ICP‐MS over the period of the study, with both agreeing with the certified value (0.710245 ± 0.000011; [39]. The measured Sr isotope ratios were corrected for instrumental fractionation by normalizing ratios to a ^86^Sr/^87^Sr ratio of 0.1194, and ^87^Rb interference was corrected using the ratio ^85^Rb/^87^Rb of 2.58745. The total procedure blank for all protocols was less than 100 pg of Sr.

**TABLE 2:** Comparison of the measured ^87^Sr/^86^Sr ratios of NIST SRM‐987 by a multi‐collector inductively coupled plasma mass spectrometer (MC‐ICP‐MS, Nu Agilent) and a thermal ionization mass spectrometer (TIMS, Thermo Fisher).

	NIST SRM‐987			
	MC‐ICP‐MS	2SD	TIMS	2SD
	0.71026	0.00001	0.71026	0.00001
	0.71023	0.00001	0.71027	0.00001
	0.71024	0.00001	0.71025	0.00001
	0.71022	0.00001	0.71025	0.00001
	0.71023	0.00001	0.71024	0.00000
	0.71024	0.00001	0.71024	0.00000
	0.71023	0.00001	0.71026	0.00000
**Av.**	**0.71024**	**0.00001**	**0.71025**	**0.00001**

## 3 Results

### 3.1 Protocol Comparisons

A comparison of the Sr isotope ratios for samples obtained by the proposed thin juice method versus the more traditional nitric acid/peroxide oxidation digestion method is shown in Table 3. Both protocols yielded identical Sr isotope ratios (within error) for sap and maple syrup samples LA1, SNA1, and 175, validating the thin juice method. These samples as well as maple syrup samples LA2, 129, and 155 analyzed by both TIMS and MC‐ICP‐MS instruments also produced similar results (within error). Thus, the thin juice method provides a rapid digestion/separation method for Sr isotopes in liquid‐based agri‐food products by either TIMS or MC‐ICP‐MS analysis.

**TABLE 3:** Comparison of the measured ^87^Sr/^86^Sr ratios of maple syrup and sap samples, with different instruments.

Sample	Type	Place	Sample preparation	MC‐ICP‐MS		TIMS		
				^87^Sr/^86^Sr	2SD	^87^Sr/^86^Sr	2SD	Difference
LA1	Sap	Laurentides	Cation exchange chromatography using a AG50X‐8 resin	0.7122	0.00003	0.7122	0.00002	0.00001
LA1	Sap	Laurentides	48 h at 80°C using HNO_3_ + H_2_O_2_	0.7121	0.00002	0.7122	0.00002	0.00000
LA2	Maple syrup	Laurentides	Cation exchange chromatography using a AG50X‐8 resin	0.7120	0.00002	0.7120	0.00002	0.00000
SNA1	Maple syrup	Saint Norbert d'Arthabaska	Cation exchange chromatography using a AG50X‐8 resin	0.7092	0.00002	0.7091	0.00002	0.00000
SNA1	Maple syrup	Saint Norbert d'Arthabaska	48 h at 80°C using HNO_3_ + H_2_O_2_	0.7092	0.00002	0.7091	0.00002	0.00000
175	Maple syrup	Les Maskoutains	Cation exchange chromatography using a AG50X‐8 resin	0.7098	0.00002	0.7098	0.00002	−0.00001
175	Maple syrup	Les Maskoutains	48 h at 80°C using HNO_3_ + H_2_O_2_	0.7098	0.00003	0.7098	0.00005	−0.00002
129	Maple syrup	Rivière‐du‐Loup	Cation exchange chromatography using a AG50X‐8 resin	0.7107	0.00001	0.7107	0.00001	0.00000
155	Maple syrup	Vaudreuil‐Soulanges	Cation exchange chromatography using a AG50X‐8 resin	0.7094	0.00001	0.7094	0.00002	0.00000

### 3.2 Soil Profile Studies

**FIGURE 3:**
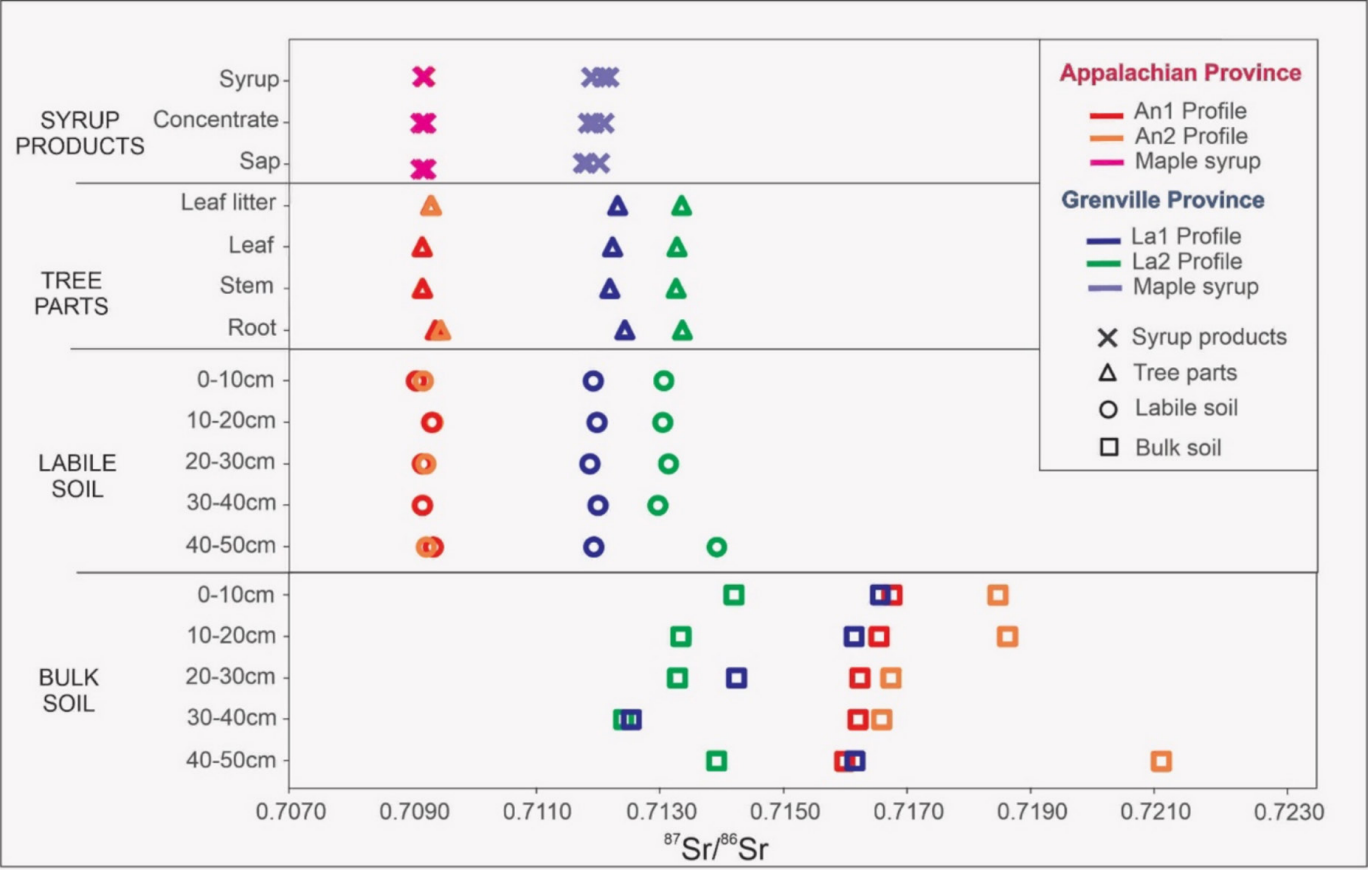
A comparison of ^87^Sr/^86^Sr values determined in bulk and labile fraction soil, maple root, stem, leaf litter, leaves, sap, concentrate sap, and syrup from Nomimingue (Laurentides) and Saint Norbert d'Arthabaska soil profiles.

Strontium isotope ratios from soil profiles and maple syrup samples are shown in Tables 4 and 5 and are plotted in Figure 3.

**TABLE 4:** Sr isotope ratios for tree samples, leaf litter, and soil profile samples.

Profile	Sample	^87^Sr/^86^Sr	2SD
La1	Leaf litter	0.7123	0.00001
	Leaves	0.7122	0.00002
	Stem	0.7122	0.00002
	Coarse roots	0.7124	0.00003
	Labile soil 0–10 cm	0.7119	0.00002
	Labile soil 10–20 cm	0.7120	0.00005
	Labile soil 20–30 cm	0.7119	0.00002
	Labile soil 30–40 cm	0.7120	0.00005
	Labile soil 40–50 cm	0.7119	0.00003
	Bulk soil 0–10 cm	0.7166	0.00001
	Bulk soil 10–20 cm	0.7161	0.00002
	Bulk soil 20–30 cm	0.7142	0.00001
	Bulk soil 30–40 cm	0.7125	0.00001
	Bulk soil 40–50 cm	0.7162	0.00001
La2	Leaf litter	0.7134	0.00001
	Leaves	0.7133	0.00001
	Stem	0.7133	0.00002
	Coarse roots	0.7134	0.00002
	Labile soil 0–10 cm	0.7131	0.00002
	Labile soil 10–20 cm	0.7130	0.00003
	Labile soil 20–30 cm	0.7131	0.00003
	Labile soil 30–40 cm	0.7130	0.00003
	Labile soil 40–50 cm	0.7139	0.00003
	Bulk Soil 0–10 cm	0.7142	0.00001
	Bulk soil 10–20 cm	0.7133	0.00002
	Bulk soil 20–30 cm	0.7133	0.00001
	Bulk soil 30–40 cm	0.7124	0.00001
	Bulk soil 40–50 cm	0.7139	0.00001
An1	Leaf litter	0.7093	0.00001
	Leaves	0.7092	0.00001
	Stem	0.7092	0.00002
	Coarse roots	0.7094	0.00002
	Labile soil 0–10 cm	0.7091	0.00003
	Labile soil 10–20 cm	0.7093	0.00002
	Labile soil 20–30 cm	0.7092	0.00002
	Labile soil 30–40 cm	0.7091	0.00004
	Labile soil 40–50 cm	0.7093	0.00004
	Bulk soil 0–10 cm	0.7168	0.00001
	Bulk soil 10–20 cm	0.7165	0.00001
	Bulk soil 20–30 cm	0.7162	0.00002
	Bulk soil 30–40 cm	0.7162	0.00004
	Bulk soil 40–50 cm	0.7160	0.00002
An2	Leaf litter	0.7093	0.00002
	Leaves	0.7092	0.00002
	Stem	0.7092	0.00001
	Coarse roots	0.7095	0.00001
	Labile soil 0–10 cm	0.7092	0.00002
	Labile soil 10–20 cm	0.7093	0.00002
	Labile soil 20–30 cm	0.7092	0.00002
	Labile soil 30–40 cm	0.7092	0.00003
	Labile soil 40–50 cm	0.7092	0.00003
	Bulk soil 0–10 cm	0.7185	0.00002
	Bulk soil 10–20 cm	0.7186	0.00002
	Bulk soil 20–30 cm	0.7167	0.00001
	Bulk soil 30–40 cm	0.7166	0.00001
	Bulk soil 40–50 cm	0.7211	0.00001

**TABLE 5:** Sr isotope ratios of maple sap products (sap, concentrate, and syrup).

		La1 and La2		An1 and An2	
Collected season	Type	^87^Sr/^86^Sr	2SD	^87^Sr/^86^Sr	2SD
Early	Sap	0.7121	0.00002	0.7092	0.00002
	Concentrate	0.7121	0.00001	0.7091	0.00001
	Syrup	0.7119	0.00001	0.7092	0.00001
1/4	Sap	0.7122	0.00003	0.7091	0.00002
	Concentrate	0.712	0.00004	0.7091	0.00001
	Syrup	0.712	0.00002	0.7092	0.00002
Mid	Sap	0.7118	0.00003	0.7092	0.00001
	Concentrate	0.7118	0.00002	0.7091	0.00001
	Syrup	0.7119	0.00002	0.7091	0.00001
3/4	Sap	0.712	0.00001	0.7091	0.00001
	Concentrate	0.7118	0.00002	0.7092	0.00002
	Syrup	0.7121	0.00001	0.7092	0.00002
Late	Sap	0.7121	0.00001	0.7092	0.00002
	Concentrate	0.7119	0.00002	0.7092	0.00001
	Syrup	0.7117	0.00002	0.7092	0.00001

In the Saint Norbert d'Arthabaska study area (An1 and An2 soil profiles), the ^87^Sr/^86^Sr isotope ratios of the bulk soils range from 0.7121 to 0.7168 and the labile fraction of the soils ranges from 0.7091 to 0.7093. The ^87^Sr/^86^Sr isotope ratios of the bulk soils are substantially more radiogenic and more variable than the ratios of the labile Sr fraction. The difference reflects the abundance of ^87^Sr in Rb‐rich silicates such as K‐Feldspar and micas that are only released through the HF‐HNO3 dissolution of the bulk soil fractions compared to the ^87^Sr‐poor phases (carbonates, loosely held Sr on mica surfaces) that are released during the labile soil extraction (see discussions in [5, 10, 12, 40, 41]).

The root, stem, leaf, and leaf litter samples yield ^87^Sr/^86^Sr ratios between 0.7092 and 0.7095. The sap, sap concentrate, and syrup sampled during different periods (early, ¼, mid, ¾, and late season) yielded very consistent values between 0.7091 and 0.7092. More importantly, the observed distribution entirely overlaps the range of values obtained from tree samples and the labile soil fraction (0.7091–0.7093).

The Sr‐isotope compositions of samples from the Laurentides region of the Grenville province (La1 and La2) are more variable. Labile soil fractions from La1 vary from 0.7119 to 0.7120 while bulk soil ratios vary from 0.7125 to 0.7166. The La2 profile yields higher ratios of 0.7130–0.7139 for the labile fraction soil and 0.7124–0.7142 for the bulk fraction soil. The different components of the maple tree show a strong correlation with the labile soil fraction, with ratios between 0.7124 and 0.7142 (La1) and 0.7124 and 0.7142 (La2). Maple syrup products, including sap and concentrate sampled during different periods of the season (early, ¼, mid, ¾, and late), vary between 0.7117 and 0.7122. ^87^Sr/^86^Sr values from the Laurentides study area are consistently higher than those from the Saint Norbert d'Arthabaska.

The large range in the labile soil fraction from the Laurentides area (0.712–0.714) suggests that the underlying geology is more variable than that in the Saint Norbert d'Arthabaska area. The heterogeneity of the labile soil values may reflect the presence of small intrusions and veins within the orthogneisses from Lacoste suite [32]. In contrast, the more limited range derived from the maple syrup samples was derived from maple syrup collected from trees over a wide area, thus representing an average of ^87^Sr/^86^Sr soil values in the producing region.

The results from all four studied profiles (Figure 3) confirm the similarity of the Sr isotope compositions of the labile soil fractions and corresponding maple trees and maple syrups. Thus, Sr isotopes are not affected by pedogenic and biologic processes, indicating that the Sr isotope composition of the labile soils can be determined by analyzing the corresponding maple products. In addition, the Sr isotope analyses of the sap, concentrate, and maple syrup products collected during early, ¼, mid, ¾, and late season of 2019 (Tables 4 and 5) yield comparable compositions. This is consistent with the observation that agrifood processing such as the fermentation of wine [13, 41, 42] or evaporation of maple syrup (present study) does not affect the ^87^Sr/^86^Sr isotopic signature of agrifood products.

### 3.3 Regional Maple Syrup Results

**FIGURE 4:**
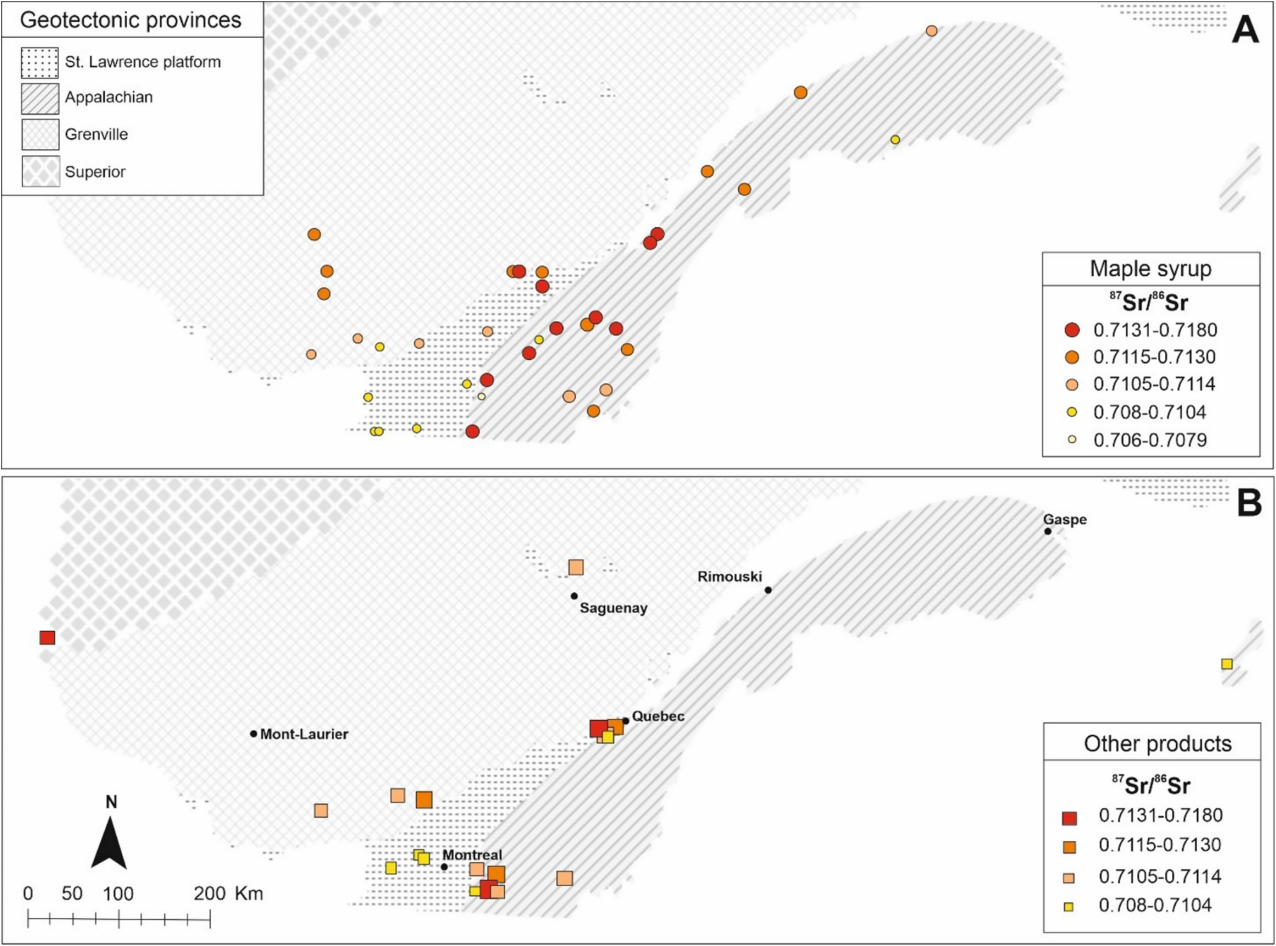
Map showing the sample type and distribution of strontium isotope ratios (^87^Sr/^86^Sr) in the province of Quebec using both present and compiled bioavailable sample results. Maple syrup sampling locations of the study are indicated (circles, A) as well as other products (cheese, milk, wine) sampling sites (squares, B). See database in Supporting Information.

The ^87^Sr/^86^Sr isotope compositions for maple syrup samples from across Quebec are presented in Table 6. The large range (from 0.7069 to 0.7177) in the ^87^Sr/^86^Sr isotope ratios of the maple syrup from across Quebec (Figure 4A) reflects the diverse ages and rock types of the three geological provinces from which the maple syrup samples were derived.

**TABLE 6:** ^87^Sr/^86^Sr results of maple syrup samples.

Province	Sample	Place	Region	^87^Sr/^86^Sr	2SD
St. Lawrence platform	L01	Vaudreuil‐Soulanges	Montérégie	0.7094	0.00001
	L02	Haut St‐Laurent	Montérégie	0.7102	0.00002
	L03	Hinchinbrooke	Hinchinbrooke	0.7102	0.00002
	L04	Les Jardins‐de‐Napierville	Montérégie	0.7099	0.00001
	L05	Les Maskoutains	Montérégie	0.7098	0.00003
	L06	St‐Alexis‐De‐Montcalm	St‐Alexis‐De‐Montcalm	0.7092	0.00001
	L07	Nicolet‐Yamaska	Centre‐du‐Québec	0.7110	0.00001
	L08	Lotbinière	Chaudière‐Appalaches	0.7161	0.00002
Appalachian	A01	Brome‐Missisquoi	Estrie	0.7168	0.00001
	A02	Roxton‐Pond	Roxton‐Pond	0.7069	0.00002
	A03	Acton	Montérégie	0.7152	0.00001
	A04	d'Arthabaska	Centre‐du‐Québec	0.7177	0.00002
	A05	Saint Norbert d'Arthabaska	Centre‐du‐Québec	0.7091	0.00002
	A06	de L'Érable	Centre‐du‐Québec	0.7168	0.00002
	A07	St‐Pierre‐De‐Broughton	St‐Pierre‐De‐Broughton	0.7143	0.00002
	A08	St‐Severin‐De‐Beauce	St‐Severin‐De‐Beauce	0.7159	0.00001
	A09	Robert‐Cliche	Chaudière‐Appalaches	0.7170	0.00002
	A10	Beauce‐Sartigan	Chaudière‐Appalaches	0.7119	0.00002
	A11	du Granit	Estrie	0.7121	0.00001
	A12	Notre‐Dame‐Des‐Bois	Notre‐Dame‐Des‐Bois	0.7127	0.00002
	A13	Haut‐Saint‐François	Estrie	0.7111	0.00001
	A14	L'Islet	Chaudière‐Appalaches	0.7174	0.00001
	A15	St‐Auber	St‐Auber	0.7171	0.00001
	A16	Rivière‐du‐Loup	Bas‐Saint‐Laurent	0.7107	0.00001
	A17	Témiscouata	Bas‐Saint‐Laurent	0.7120	0.00002
	A18	La Mitis	Bas‐Saint‐Laurent	0.7124	0.00002
	A19	Marsoui	Marsoui	0.7102	0.00001
	A20	d'Avignon	Gaspésie–Îles‐de‐la‐Madeleine	0.7098	0.00001
Grenville	G01	Antoine‐Labelle	Laurentides	0.7121	0.00001
	G02	Laurentides	Laurentides	0.7121	0.00002
	G03	Nominingue	Nominingue	0.7124	0.00002
	G04	Papineau	l'Outaouais	0.7105	0.00002
	G05	Montcalm	Lanaudière	0.7102	0.00002
	G06	Montcalm	Lanaudière	0.7092	0.00001
	G07	Pays‐d'en‐Haut	Laurentides	0.7085	0.00001
	G08	L'avenir	L'avenir	0.7116	0.00002
	G09	St‐Ubalde	St‐Ubalde	0.7149	0.00002
	G10	Portneuf	Capitale‐Nationale	0.7102	0.00001
	G11	Portneuf	Capitale‐Nationale	0.7111	0.00002

For example, the lowest ^87^Sr/^86^Sr ratio measured among the maple syrup samples was derived from a maple bush on the flanks of an intermediate to ultramafic Cretaceous alkaline intrusion in the Roxton‐Pond region, emplaced along the border of the Appalachian and St. Lawrence platform geological provinces.

In the Grenville province, the highest ^87^Sr/^86^Sr isotope composition (0.7149) is related to paragneiss rocks in the region of St‐Ubalde, while the lowest ^87^Sr/^86^Sr isotope composition (0.7085) is derived from a maple bush overlying a meta‐sedimentary sequence consisting of marble, calc‐silicate rocks, dolomite, quartz feldspathic schists, and quartzite from the Pays‐d'en‐Haut area. Maple syrup samples derived from granite and orthogneiss terrains within the Grenville province generally show a lower degree of variability in their ^87^Sr/^86^Sr isotope compositions (from 0.7102 to 0.7124). The samples from the Montcalm and Portneuf maple groves are derived from regions that straddle both the Grenville province and the St. Lawrence Platform; thus, the variation in their Sr isotope compositions (0.7092–0.7111) may reflect the geology of both provinces.

Appalachian province samples display values ranging from 0.7091 to 0.7177, reflecting the different geological features of the sampled terrains. The lowest value is associated with limestone‐rich terrains from Saint Norbert d'Arthabaska Centre‐du‐Québec as well as clay‐limestones sequences from d'Avignon, Gaspésie, and les Îles‐de‐la‐Madeleine (0.7098). The highest values are associated with a bedrock consisting of mica‐rich schists and metapelites that characterize the central portion of the Appalachian province.

In contrast to the above provinces, the variation in ^87^Sr/^86^Sr isotope compositions for maple samples from the St Lawrence platform is relatively low (0.7092–0.7110). The Lotbinière maple syrup value (0.7162) can be considered an outlier. The bedrock in this region consists of Ordovician mudrocks and shales of the Lorraine Group. The higher ^87^Sr/^86^Sr ratio could reflect the clay‐rich nature of the Lorraine Group.

## 4 Discussion

The Sr isotope data for the maple syrup analyses that are plotted on the map in Figure 4a are compared with the locations of previously studied agrifood products such as cheese, milk, and wine (Figure 4B). There is a general similarity in the range of Sr isotope ratios in a given geological province for the different agrifood products.

### 4.1 Bioavailable ^87^Sr/^86^Sr Map of Quebec

**FIGURE 5:**
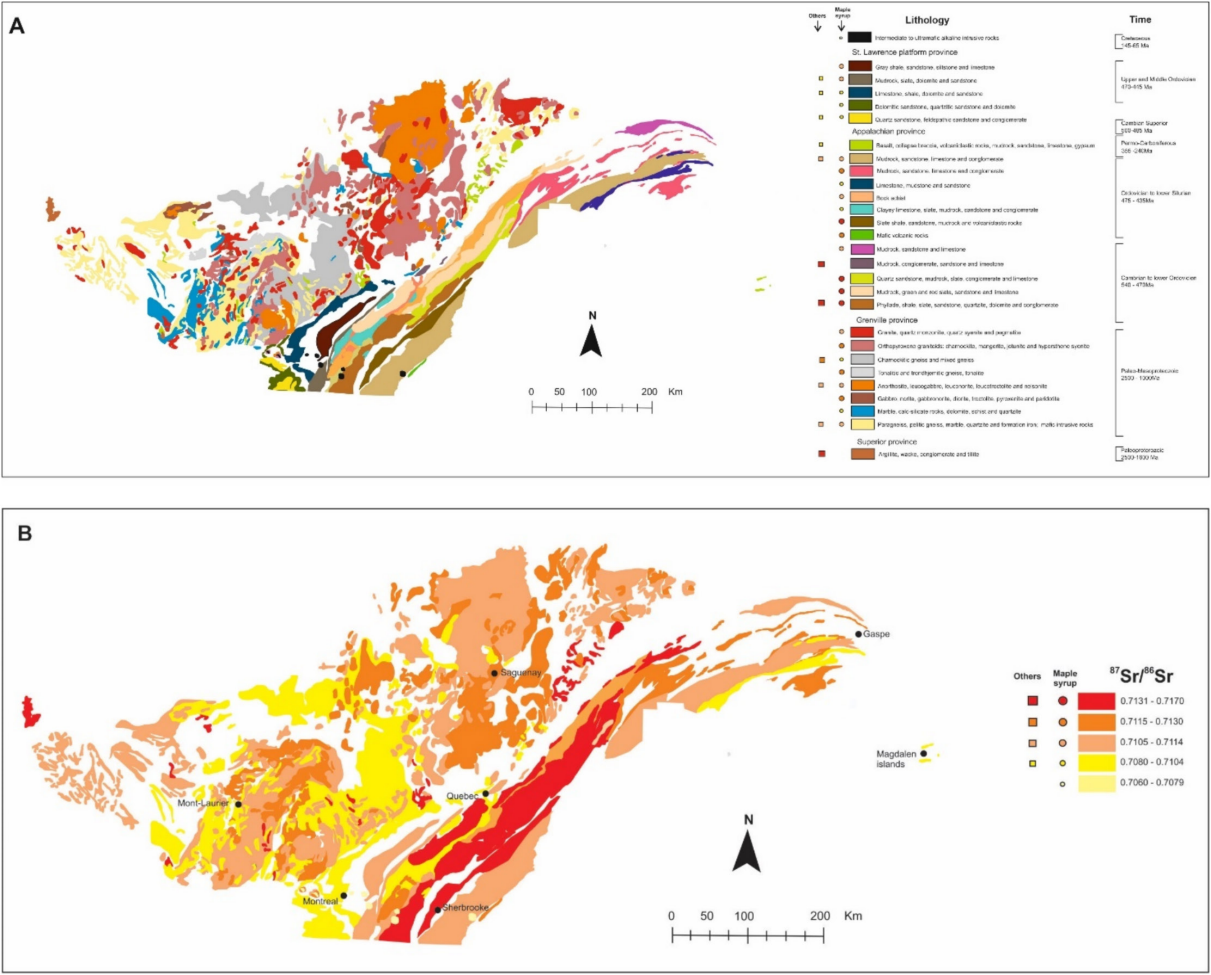
(A) Lithological map of Quebec showing units with bioavailable ^87^Sr/^86^Sr results. The presented lithologies represent all units with ^87^Sr/^86^Sr information analyzed in this work or included from the literature. Agrifood ^87^Sr/^86^Sr values were extrapolated to the same rock units; (B) ^87^Sr/^86^Sr Isoscape showing the evolution of bioavailable strontium ratios into Superior, Grenville, Appalachian, and St. Lawrence platform geotectonic provinces individualized by period and lithology. Maps developed in ArcGIS Pro using the geological database from Quebec province [14].

Strontium bioavailable mapping (isoscapes) uses the present‐day ^87^Sr/^86^Sr ratios of bedrock, soils, and plants to create a map in combination with geological data [43]. Bioavailable ^87^Sr/^86^Sr maps of varying types of samples, scales, and resolutions have been globally published for traceability purposes, in small or large scales [40]. Combining lithological and bioavailable maps provides a means of estimating the bioavailable ^87^Sr/^86^Sr; however, there are advantages and limitations of mapping bioavailable ^87^Sr/^86^Sr and bedrock background for provenance studies [41, 42, 43]. Authors (see reviews by [44]) have noted the indiscriminate use of isotopic data without the evaluation of types of sampled materials (rock, labile soils, ground water, plants, tooth, bone, wine, etc.). [45] concluded that small animals, plants, and soils are the preferred types of samples to develop ^87^Sr/^86^Sr isoscape maps. In light of this consensus, we combined the regional Sr isotope data for the maple syrup analyses of this study with previously published agrifood Sr isotope studies in Quebec (data in Table S1) to construct a bioavailable Sr isotope map for the province (Figure 5). The Sr isotope data was incorporated in ESRI ArcGIS Pro 3.0.0 using the Spatial Analyst and Geostatistical Analyst extensions coupled with a geological map of Quebec (1:2000.000, [14]).

The large sampling density, availability of high‐quality lithological maps, and proven strong correlation of ^87^Sr/^86^Sr values between rock, soil, labile soil, and agrifood products [10, 13, 18] lend themselves well to the production of an isocape. The isoscape provides context for future Sr isotope agrifood studies for terroir characterization and Sr isotope studies for biometric tracing of human and fauna migration.

In Figure 5A, we selected 26 lithological units that presented one or more Sr isotope results. Similar ^87^Sr/^86^Sr ratios for either maple syrup, tree roots, or other agricultural samples (milk, cheese) from the same geological unit underscore the geological dependence of the bioavailable Sr and the integrity of the ^87^Sr/^86^Sr isoscape maps. However, in the 26 different lithologies investigated, overlapping ^87^Sr/^86^Sr ratios were found in 8 of the lithological units, indicating that the strontium isotope ratios are not exclusive to a given geological unit.

In Figure 5B, the lithological units underlying the agrifood products from the province of Quebec are color‐coded into 5 different isotope groups based on spatial analysis (see Supporting Information) and represent the first bioavailable ^87^Sr/^86^Sr map for Canada.

The map reveals that lithologies with the highest Sr isotope ratios (0.7131–0.7177) are predominantly associated with mudrocks that are typically enriched in ^87^Sr due to their Rb‐rich compositions. Examples of these types of rocks are found in Cambrian to Ordovician (540–470 Ma) rocks in the Appalachian Province. Spatial patterns of ^87^Sr/^86^Sr results from Rb‐rich rocks are pronounced, following the regional geology, and elongated along a NE–SW direction, with higher ^87^Sr/^86^Sr in the central part of the map. These rocks encompass an age range between 550 and 435 Ma and are systematically higher than those of the adjoining Saint Lawrence platform (470–450 Ma). This distribution indicates that the ^87^Sr/^86^Sr results are not necessarily associated with a specific province or age, but with rock composition (Figure 5). For example, the lowest Sr isotope ratios are related to maple syrup samples from Sr‐rich carbonate rocks in the Grenville (0.7085), Appalachian (0.7090) and Saint Lawrence platform (0.7092) provinces. This first bioavailable ^87^Sr/^86^Sr map of Quebec is encouraging, but due to the high complexity of the geology, a clearer discrimination of ^87^Sr/^86^Sr distribution can be obtained by densifying and diversifying sample collections and geological unities.

## 5 Conclusions

This paper reported an improved method for the separation of strontium for ^87^Sr/^86^Sr isotope ratio studies in liquids by combining two different cation‐exchange chromatography steps. Results confirm that ^87^Sr/^86^Sr maple syrup methodology can be considered a reliable protocol for use in agri‐food provenance studies.

This study demonstrated that there is no fractionation of the ^87^Sr/^86^Sr ratio between the labile soil fraction, different parts of the maple tree, and all products from maple sap (sap, concentrate and maple syrup). We also did not notice differences during the different phases of evaporation of the sap to produce maple syrup. We confirm that the underlying geology is the dominant control on the isotope composition across different geological provinces in Quebec. The study demonstrates that the ^87^Sr/^86^Sr ratios of maple syrup are faithful to the underlying geology and that the ^87^Sr/^86^Sr ratios can be a powerful tracer of provenance at the local scale with local geological control.

The data from this study was used to develop a bioavailable ^87^Sr/^86^Sr map of Quebec that can be used to predict the geographical origin of agrifood products from southern Quebec. The map is a promising first step given the relatively small density of samples. Further sampling is needed to establish the robustness of the method and the map.

## Author Contributions

**R. Stevenson:** conceptualization, investigation, funding acquisition, methodology, validation, visualization, writing – review and editing, supervision. **M. Sadiki:** conceptualization, investigation, funding acquisition, writing – review and editing, methodology, validation, resources. **L. Lagacé:** conceptualization, investigation, funding acquisition, methodology, validation, writing – review and editing, resources. **D. Widory:** conceptualization, investigation, funding acquisition, methodology, validation, writing – review and editing, supervision, resources, project administration.

## Supporting information


**Table S1.** Agrifood sample type and ^87^Sr/^86^Sr ratios available in the literature for the province of Quebec. Sample coordinates are reported in the World Geodetic System 1984 (WGS84).

**Table S2.** 87Sr/86Sr isotopic data for agrifood samples from Quebec.

## Data Availability

The data that support the findings of this study are available from the corresponding author upon reasonable request.
