# Supplementary material for: An Improved Chemical Extraction Procedure for the Sr Isotope Analysis of Liquid Agrifood Samples Applied to Authenticating the Origin of Maple Syrups in Quebec (Canada)
Source: Rapid Commun Mass Spectrom. 2025 May 13;39(16):e10066. doi: 10.1002/rcm.10066 (PMC12075995; doi:10.1002/rcm.10066)
Supplement: Supplementary file 1 — Table S1. Agrifood sample type and 87Sr/86Sr ratios available in the literature for the province of Quebec. Sample coordinates are reported in the World Geodetic System 1984 (WGS84). [file RCM-39-e10066-s001.docx]

Supporting Material

**An improved chemical extraction procedure for the Sr isotope analysis of liquid agrifood samples applied to authenticating the origin of maple syrups in Quebec (Canada)**

B. Saar de Almeida^1^, R. Stevenson^1^, M. Sadiki^2^, L. Lagacé^2^, D. Widory^1^

^1^ Université du Québec à Montréal (UQAM)/GEOTOP, Ave du Président Kennedy, Montréal, QC, Canada

^2^ Centre de recherche, de développement et de transfert technologique acéricole Inc. (Centre ACER), 142 rang Lainesse, St-Norbert d’Arthabaska, QC, Canada

Corresponding author: B. Saar de Almeida; saar_de_almeida.bruna@uqam.ca.

**Supporting information includes:** 1 Table.

Table S1. Agrifood sample type and ^87^Sr/^86^Sr ratios available in the literature for the province of Quebec. Sample coordinates are reported in the World Geodetic System 1984 (WGS84).

| X | Y | Product | Type | ^87^Sr/^86^Sr | 2SD | Reference |
| --- | --- | --- | --- | --- | --- | --- |
| 47.126773 | -70.495985 | Cheese | Cow | 0.7130 | 0.000040 | Stevenson et al., 2015 |
| 47.126773 | -70.495985 | Cheese | Cow | 0.7140 | 0.000040 | Stevenson et al., 2015 |
| 48.548802 | -71.653571 | Cheese | Cow | 0.7110 | 0.000010 | Stevenson et al., 2015 |
| 48.548802 | -71.653571 | Cheese | Cow | 0.7110 | 0.000040 | Stevenson et al., 2015 |
| 45.415362 | -71.623819 | Cheese | Goat | 0.7100 | 0.000030 | Stevenson et al., 2015 |
| 45.415362 | -71.623819 | Cheese | Goat | 0.7100 | 0.000020 | Stevenson et al., 2015 |
| 46.125423 | -73.701745 | Cheese | Goat | 0.7120 | 0.000030 | Stevenson et al., 2015 |
| 46.125423 | -73.701745 | Cheese | Goat | 0.7110 | 0.000030 | Stevenson et al., 2015 |
| 47.378332 | -61.912452 | Cheese | Cow | 0.7090 | 0.000020 | Stevenson et al., 2015 |
| 47.378332 | -61.912452 | Cheese | Cow | 0.7100 | 0.000100 | Stevenson et al., 2015 |
| 47.330486 | -79.435922 | Cheese | Cow | 0.7130 | 0.000010 | Stevenson et al., 2015 |
| 47.330486 | -79.435922 | Cheese | Cow | 0.7130 | 0.000030 | Stevenson et al., 2015 |
| 45.416708 | -74.096598 | Grape | Sabrevois | 0.7090 | 0.000020 | Vinciguerra et al., 2016 |
| 46.931858 | -71.012348 | Grape | Sainte Croix | 0.7100 | 0.000020 | Vinciguerra et al., 2016 |
| 46.931858 | -71.012348 | Grape | Maréchal Foch | 0.7090 | 0.000030 | Vinciguerra et al., 2016 |
| 45.468892 | -72.884336 | Grape | Marquette | 0.7090 | 0.000010 | Vinciguerra et al., 2016 |
| 45.455394 | -72.896711 | Grape | Sainte Croix | 0.7100 | 0.000010 | Vinciguerra et al., 2016 |
| 45.452336 | -72.890585 | Grape | Marquette | 0.7100 | 0.000020 | Vinciguerra et al., 2016 |
| 45.416165 | -72.608872 | Grape | Sabrevois | 0.7130 | 0.000020 | Vinciguerra et al., 2016 |
| 45.262406 | -72.589130 | Grape | Maréchal Foch | 0.7100 | 0.000160 | Vinciguerra et al., 2016 |
| 45.253715 | -72.713270 | Grape | Sabrevois | 0.7140 | 0.000020 | Vinciguerra et al., 2016 |
| 45.233166 | -72.854569 | Grape | Marquette | 0.7100 | 0.000020 | Vinciguerra et al., 2016 |
| 46.927232 | -71.072971 | Grape | Maréchal Foch | 0.7090 | 0.000030 | Vinciguerra et al., 2016 |
| 46.864748 | -71.117000 | Grape | Sainte Croix | 0.7120 | 0.001150 | Vinciguerra et al., 2016 |
| 46.864748 | -71.117000 | Grape | Frontenac | 0.7120 | 0.000020 | Vinciguerra et al., 2016 |
| 46.885192 | -71.080797 | Grape | Marquette | 0.7150 | 0.000010 | Vinciguerra et al., 2016 |
| 46.885192 | -71.080797 | Grape | Frontenac | 0.7130 | 0.000030 | Vinciguerra et al., 2016 |
| 46.965428 | -70.968228 | Grape | Marquette | 0.7120 | 0.000020 | Vinciguerra et al., 2016 |
| 45.452336 | -72.890585 | Grape | Marquette | 0.7100 | 0.000004 | Guibourdenche et al., 2020 |
| 45.262406 | -72.589130 | Grape | Maréchal Foch | 0.7100 | 0.000009 | Guibourdenche et al., 2020 |
| 45.416165 | -72.608872 | Grape | Sabrevois | 0.7130 | 0.000004 | Guibourdenche et al., 2020 |
| 45.253715 | -72.713270 | Grape | Sabrevois | 0.7140 | 0.000003 | Guibourdenche et al., 2020 |
| 45.253715 | -72.713270 | Grape | Sabrevois | 0.7140 | 0.000004 | Guibourdenche et al., 2020 |
| 45.468892 | -72.884336 | Grape pulp | Marquette | 0.7100 | 0.000009 | Guibourdenche et al., 2020 |
| 45.452336 | -72.890585 | Grape pulp | Marquette | 0.7100 | 0.000007 | Guibourdenche et al., 2020 |
| 45.262406 | -72.589130 | Grape pulp | Maréchal Foch | 0.7100 | 0.000008 | Guibourdenche et al., 2020 |
| 45.416165 | -72.608872 | Grape pulp | Sabrevois | 0.7120 | 0.000007 | Guibourdenche et al., 2020 |
| 45.253715 | -72.713270 | Grape pulp | Sabrevois | 0.7140 | 0.000007 | Guibourdenche et al., 2020 |
| 45.468892 | -72.884336 | Grape seed | Marquette | 0.7102 | 0.000010 | Guibourdenche et al., 2020 |
| 45.452336 | -72.890585 | Grape seed | Marquette | 0.7101 | 0.000010 | Guibourdenche et al., 2020 |
| 45.262406 | -72.589130 | Grape seed | Maréchal Foch | 0.7106 | 0.000009 | Guibourdenche et al., 2020 |
| 45.416165 | -72.608872 | Grape seed | Sabrevois | 0.7133 | 0.000007 | Guibourdenche et al., 2020 |
| 45.253715 | -72.713270 | Grape seed | Sabrevois | 0.7144 | 0.000013 | Guibourdenche et al., 2020 |
| 45.468892 | -72.884336 | Grape skin | Marquette | 0.7102 | 0.000009 | Guibourdenche et al., 2020 |
| 45.452336 | -72.890585 | Grape skin | Marquette | 0.7101 | 0.000013 | Guibourdenche et al., 2020 |
| 45.262406 | -72.589130 | Grape skin | Maréchal Foch | 0.7106 | 0.000008 | Guibourdenche et al., 2020 |
| 45.416165 | -72.608872 | Grape skin | Sabrevois | 0.7134 | 0.000007 | Guibourdenche et al., 2020 |
| 45.253715 | -72.713270 | Grape skin | Sabrevois | 0.7145 | 0.000018 | Guibourdenche et al., 2020 |
| 45.468892 | -72.884336 | Grapes | Marquette | 0.7102 | 0.000004 | Guibourdenche et al., 2020 |
| 46.931858 | -71.012348 | Labile soil | Labile Soil | 0.7121 | 0.000020 | Vinciguerra et al., 2016 |
| 46.923624 | -71.248072 | Maple wood | Maple Tree | 0.7135 | 0.000400 | Houle et al., 2021 |
| 46.149166 | -74.079722 | Maple wood | Maple Tree | 0.7101 | 0.000400 | Houle et al., 2021 |
| 45.931884 | -75.164452 | Maple wood | Maple Tree | 0.7108 | 0.001200 | Houle et al., 2021 |
| 47.126773 | -70.495985 | Milk | Cow | 0.7143 | 0.000010 | Stevenson et al., 2015 |
| 47.126773 | -70.495985 | Milk | Cow | 0.7142 | 0.000030 | Stevenson et al., 2015 |
| 48.548802 | -71.653571 | Milk | Cow | 0.7114 | 0.000010 | Stevenson et al., 2015 |
| 48.548802 | -71.653571 | Milk | Cow | 0.7117 | 0.000020 | Stevenson et al., 2015 |
| 45.415362 | -71.623819 | Milk | Goat | 0.7110 | 0.000010 | Stevenson et al., 2015 |
| 45.415362 | -71.623819 | Milk | Goat | 0.7108 | 0.000040 | Stevenson et al., 2015 |
| 46.125423 | -73.701745 | Milk | Goat | 0.7117 | 0.000020 | Stevenson et al., 2015 |
| 46.125423 | -73.701745 | Milk | Goat | 0.7121 | 0.000100 | Stevenson et al., 2015 |
| 47.378332 | -61.912452 | Milk | Cow | 0.7096 | 0.000010 | Stevenson et al., 2015 |
| 47.378332 | -61.912452 | Milk | Cow | 0.7097 | 0.000030 | Stevenson et al., 2015 |
| 47.330486 | -79.435922 | Milk | Cow | 0.7135 | 0.000010 | Stevenson et al., 2015 |
| 47.330486 | -79.435922 | Milk | Cow | 0.7130 | 0.000020 | Stevenson et al., 2015 |
| 45.416708 | -74.096598 | Wine | Sabrevois | 0.7102 | 0.000010 | Vinciguerra et al., 2016 |
| 46.931858 | -71.012348 | Wine | Sainte Croix | 0.7108 | 0.000010 | Vinciguerra et al., 2016 |
| 46.931858 | -71.012348 | Wine | Maréchal Foch | 0.7099 | 0.000010 | Vinciguerra et al., 2016 |
| 45.468892 | -72.884336 | Wine | Marquette | 0.7101 | 0.000010 | Vinciguerra et al., 2016 |
| 45.455394 | -72.896711 | Wine | Sainte Croix | 0.7114 | 0.000010 | Vinciguerra et al., 2016 |
| 45.452336 | -72.890585 | Wine | Marquette | 0.7102 | 0.000010 | Vinciguerra et al., 2016 |
| 45.416165 | -72.608872 | Wine | Sabrevois | 0.7131 | 0.000020 | Vinciguerra et al., 2016 |
| 45.262406 | -72.589130 | Wine | Maréchal Foch | 0.7111 | 0.000010 | Vinciguerra et al., 2016 |
| 45.253715 | -72.713270 | Wine | Sabrevois | 0.7144 | 0.000030 | Vinciguerra et al., 2016 |
| 45.233166 | -72.854569 | Wine | Marquette | 0.7107 | 0.000020 | Vinciguerra et al., 2016 |
| 46.927232 | -71.072971 | Wine | Maréchal Foch | 0.7102 | 0.000020 | Vinciguerra et al., 2016 |
| 46.864748 | -71.117000 | Wine | Sainte Croix | 0.7140 | 0.000020 | Vinciguerra et al., 2016 |
| 46.864748 | -71.117000 | Wine | Frontenac | 0.7131 | 0.000020 | Vinciguerra et al., 2016 |
| 46.885192 | -71.080797 | Wine | Marquette | 0.7151 | 0.000020 | Vinciguerra et al., 2016 |
| 46.885192 | -71.080797 | Wine | Frontenac | 0.7135 | 0.000020 | Vinciguerra et al., 2016 |
| 46.885192 | -71.080797 | Wine | Maréchal Foch | 0.7155 | 0.000010 | Vinciguerra et al., 2016 |
| 46.965428 | -70.968228 | Wine | Marquette | 0.7121 | 0.000010 | Vinciguerra et al., 2016 |
| 45.262406 | -72.589130 | Wine | Maréchal Foch | 0.7111 | 0.000006 | Guibourdenche et al., 2020 |
| 45.262406 | -72.589130 | Wine | Maréchal Foch | 0.7113 | 0.000005 | Guibourdenche et al., 2020 |
| 45.416165 | -72.608872 | Wine | Sabrevois | 0.7130 | 0.000004 | Guibourdenche et al., 2020 |
| 45.253715 | -72.713270 | Wine | Sabrevois | 0.7143 | 0.000004 | Guibourdenche et al., 2020 |
| 45.253715 | -72.713270 | Wine | Sabrevois | 0.7143 | 0.000006 | Guibourdenche et al., 2020 |
| 45.468892 | -72.884336 | Wine | Marquette | 0.7102 | 0.000006 | Guibourdenche et al., 2020 |
| 45.452336 | -72.890585 | Wine | Marquette | 0.7102 | 0.000005 | Guibourdenche et al., 2020 |
| 45.452336 | -72.890585 | Wine | Marquette | 0.7102 | 0.000004 | Guibourdenche et al., 2020 |
